# Monthly Summary

**Published:** 1868-03

**Authors:** 


### MONTHLY SUMMARY.

---

*Muscular Contraction.*—The advocates of the old doctrine that muscle is destroyed during contraction, and that the urea eliminated is the measure of this destruction, were confounded by the famous experiments of Fick and Wislicenus. Those experiments appeared to prove that the true source of muscular activity was non-nitrogenous food. Dr. Parker has taken up the interesting question and has studied the phenomena in two soldiers.

Without going into details, suffice it to say, that with the same quantity of ingested nitrogen, there was an increased excretion of azotized matter when the voluntary muscles were at rest, and that, on the contrary, in opposition to all our preconceived notions, this excretion was diminished during periods of active exercise. When rest, however, followed a period of active exercise, there was a slight but long-continued excess of nitrogenous excretions. If the supply of nitrogen was cut off for any length of time, there was a retention of this element in the system when it was again supplied by food, showing that a temporarily insufficient supply must be subsequently compensated.

Now, there is waste going on throughout the tissues. Gland cells, bones, nerves, tendons, all supply this current. The involuntary muscles too, are constantly eliminating it, and all this must be taken into consideration when we study the excretion of urea. As, however, this quantity is in a measure constant, we are left to account for the proportions excreted.

Dr. Parker suggests that exercise enables the muscles to absorb and appropriate more nitrogen from the general store of the blood, and that during rest they eliminate this element. A more abundant absorption would therefore be followed by a more copious excretion during the subsequent period of rest. This opinion, which seems justified by the experiments, changes the whole elements of the calculation. If correct, we must estimate muscular action not by the amount of nitrogen eliminated but by the quantity absorbed, if indeed, we ever can get at the latter.

"When a voluntary muscle" says Dr. Parker, "is brought into action by the influence of the will, it appropriates nitrogen and grows; the stimulus in the act of union gives rise to changes in the non-nitrogenous substances surrounding the ultimate elements of the muscular substance, which cause the conversion of heat into motion. The contraction continues (the will still acting) until the effete products of these changes avert it; a state of rest ensues, during which time the effete products are removed, the muscle loses nitrogen, and can again be called into action by its stimulus."

The theory is certainly ingenious and merits close and attentive investigation.

---

*Cholera and Crocodiles.*—A French writer maintains that the prevalence of cholera in India is due to the disappearance of the gavial, a species of crocodile native to the Ganges. This creature, he maintains, used to eat the bodies of the Indians, thrown into the sacred river by the superstitious devotees inhabiting its banks. The soldiers, however, have amused themselves with hunting this useful creature, which is now nearly extinct. The holy stream, missing its scavengers, revenges itself upon the slayers by spreading cholera far and wide. The writer asks government protection for the valuable reptile.

---

*A New Styptic.*—Perchloride of iron, combined with collodion is a good hæmostatic for wounds, the bite of leeches, etc.

To prepare it, one part of crystallized perchloride of iron is mixed with six parts of collodion. The perchloride of iron should be added gradually and carefully, to prevent the evolution of excessive heat, which injures the collodion. The composition, when well made, is of a yellowish red color, perfectly limpid, and produces on the skin a yellow pellicle which retains great elasticity.

---

*Mental Labour and Physical Exertion.*—It may be possible, at some future stage of scientific enquiry, to compute the comparative amount of oxidation in the brain during severe mental labor. Even now, from obvious facts, we must pronounce it to be a very considerable fraction of the entire work done in the system. The privation of the other interests during mental exertion is so apparent, so extensive, that if the exertion should happen tc be long continued, a liberal atonement has to be made in order to stave off general insolvency. Mental excess counts as largely as muscular excess in the diversion of power: it would be competent to suppose either the one or the other reducing the remaining forces of the system to one-half of their proper amount. In both cases the work of restoration must be on the same simple plan of redressing the inequality by allowing more than the average flow of blood to the impoverished organs, for a length of time corresponding to the period when their nourishment has been too small. It is in this consideration that we seem to have the reasonable, I may say the arithmetical, basis of the constitutional treatment of chronic disease. We repay the debt to nature by allowing the weakened organ to be better nourished and less taxed, according to the degradation it has undergone by the opposite line of treatment. In a large class of diseases we have obviously a species of insolvency, to be dealt with according to the sound method of readjusting the relations of expenditure and income. And if such be the true theory, it seems to follow that medication is only an inferior adjunct. Drugs, even in the happiest application, can but guide and favor the restorative process; just as the stirring of a fire may make it burn, provided there be the needful fuel. There is thus a definite, although not numerically stateable, relation between the total of the physico-mental forces and the total of the purely physical processes. The grand aggregate of the oxidation of the system includes both; and the more the force taken up by one, the less is left to the other. Such is the statement of the correlation of mind to the other forces of nature. We do not deal with pure mind—mind in the abstract; we have no experience of an entity of that description. We deal with a compound or two-sided phenomenon—mental on one side, physical on the other; there is a definite correspondence in degree, although a difference of nature, between the two sides; and the physical side is itself in full correlation with the recognized physical forces of the world.—*Macmillian's Magazine.*

---

*To Prevent Pitting from Small Pox.*—It has long been known that the exclusion of light tends to diminish the effect of small pox in marking the face. Those parts of the body from which light is excluded by the clothing, present no permanent marks. Various devices have been contrived to exclude both light and air from the face in the eruptive stage—such as smearing with lard and ointments—covering with a mask, and so forth. Dr. Black, of London, (*Lancet*) considers nothing more to be required than to exclude light. In several cases he smeared the face with lard and kept the patients in absolute darkness, giving at the same time, in the iniatory stage, liq. ammon. acet. and liq. potass. arsen. one or two drachms of the former, and three drops of the latter, every three hours. After the acumination of the pustules, the liq. ammon. was suspended, and four or five drops of dilute nitric acid substituted, the arsenic being continued. His theory is that the variolous poison in the blood is neutralized in some degree by the arsenite of potassa; that the ammonia promotes the action of the skin, and that the acid promotes desiccation. Though he attributes the absence of pitting to the exclusion of light, yet he regards the internal treatment as important in preventing or moderating the fever.—*Pacific Med. and Surg. Journal.*

---

*Compression of Carotids for Convulsions.*—Some curious results of this treatment are given by a French practitioner, M. Favez. He relates three cases of convulsions in which it was successful. The first was that of a child, æt. 6 years, who had violent spasms of the left side of the body, with clenched jaws, bitten tongue, etc. Compression of the *right* carotid stopped the fit immediately; the child fell asleep and awoke in full consciousness a quarter of an hour afterwards. The second was a girl of 7 years. She had convulsions of the right side of the body, apparently produced by fright. Here compression of the *left* carotid produced equally happy results. The third was a child of 2½ years, with convulsions of *both* sides. Compression of the right carotid at once arrested the movements of the left side. The left carotid was then compressed and the convulsions of the right side ceased. Sleep followed, and the patient awoke in an hour, quite well.—*Pacific Med. and Surg. Journal.*

---

*The Human Voice.*—Dion Boucicault, commenting on the Albert Hall of Science and Art, in the *Pall Mall Gazette*, says: "The human voice, when speaking with clear articulation and applied from good lungs, will fill 400,000 cubic feet of air, provided they be enclosed in a proper manner, and the voice placed and directed advantageously. The same voice singing can fill, with equal facility, 600,000 cubic feet. When singing, the vowels are principally used, because it is necessary to dwell upon a note, and we cannot prolong a consonant. In speaking, on the contrary, we depend for articulation on the consonants; but their short percussive sound does not travel. When we shout, or speak in the open-air which partakes of shouting, we prolong the vowels, drawling the syllable of each word; but what we gain in sound we lose in clearness of articulation; expression is lost in monotony, because its fineness depends on the infinite variety of which the consonant is capable and bestows on the vowel. Two thousand voices, singing or speaking together, travel no further than one voice. They may fill a certain area more completely with that intricacy of waves which, when very troublesome, we call a din; but each voice exerts its own influence on the air, according to its power, and dies away within certain limits. A second voice acts independently, and produces its own separate effect, not fortifying the first, but distinct from it. And so with any number of voices—say 10,000—shouting together; if a single trumpeter were placed among them, the note of his trumpet would be heard clearly at a distance where the Babel of voices would have expired in a murmer. Yet among the din produced by the 10,000 voices, the trumpet would be inaudible. To illustrate this theory more clearly, it is plain that 2,000 persons cannot throw stones further than one person. It is true that the air, within certain limits, will be more full of stones; but they will all come to the ground within a limited area.—*Druggists' Circular.*

